# Giant composite pheochromocytoma and gastrointestinal stromal tumor in a patient with neurofibromatosis: A case report

**DOI:** 10.14744/nci.2020.37431

**Published:** 2021-12-29

**Authors:** Soykan Arikan, Cihad Tatar, Ali Emre Nayci, Feyzullah Ersoz, Mehmet Baki Dogan, Feray Gunver

**Affiliations:** 1. Department of General Surgery, Istanbul Training and Research Hospital, Istanbul, Turkey; 2. Department of Pathology, Istanbul Training and Research Hospital, Istanbul, Turkey

**Keywords:** Gastrointestinal stromal tumor, pheochromocytoma, neurofibromatosis

## Abstract

A 54-year-old male was admitted to our department with neurofibromatosis and hypertension. During his examination, a mass was detected in the abdomen, and he was transferred to a surgical clinic. At the first examination of the patient, extensive café-au-lait spots and granulomas were detected on the body and the mass occupying right abdomen quadrant was palpable. The patient’s medical history indicated that he had hypertension for almost a decade. The patient also stated that nodules on the body existed from his earliest recollection and he had relatives with neurofibromatosis. The patient was taken to a surgical operation. A mass with 30×23 cm in size was removed. The area of the nodular structure, with 0.5 cm in diameter, in the stomach serosa was also removed. The tumor was composed of phaeochromocytoma in the larger spaces and ganglioneuromas in the relatively narrow spaces. The nodular area removed in gastric serosa was reported as a very low-risk gastrointestinal stromal tumor. Apart from this rare combination, adrenal mass removed from the patient was considerably larger than the masses in the literature until now. Therefore, we aimed to present this rare case with a literature background.

**N**eurofibromatosis is a common disease that displays an autosomal dominant pattern of inheritance. The prevalence of neurofibromatosis is one in 3000 live births in Western countries [1]. It is possible to observe neurofibromatosis, also known as Von Recklinghausen disease, in association with various disorders such as; neoplastic lesions such as neurofibromas, malignant peripheral nerve sheath tumors, carcinoid tumors, gastrointestinal stromal tumors (GIST), and pheochromocytoma. Among these lesions that are spanned a wide range of malignant or benign appearances in neurofibromatosis patients, some of them might be observed to be located in the abdominal area [2, 3].

Pheochromocytoma, one of the aforementioned lesions, is found in approximately 1% of patients with neurofibromatosis. Moreover, in some cases, pheochromocytoma is present as composite tumors in which neuroblastoma, ganglioneuroblastoma, or ganglioneuroma are coexisted with pheochromocytoma.

Although GISTs are the most common mesenchymal neoplasm of the gastrointestinal tract, they are classified as tumors with a rare incidence. They are also closely related to neurofibromatosis. These three disorders are very scarcely developed within a patient, although it was previously reported for a few times in the literature [4].

Neurofibromatosis, composite pheochromocytoma with ganglioneuroma, and GIST were detected in a patient who was under treatment in our clinic. Apart from this rare combination, adrenal mass removed from the patient was considerably larger than the masses in the literature until now. It is rare to identify both a GIST and a pheochromocytoma in a patient with NF1. Therefore, we aimed to present this rare case with a literature background.

## Case Report

A 54-year-old male was admitted to our department with neurofibromatosis and hypertension. During his examination, a mass was detected in the abdomen and he was transferred to the surgical clinic. At the first examination of the patient, extensive café-au-lait spots and granulomas were detected on the body and the mass occupying right abdomen quadrant was palpable.

The patient’s medical history indicated that he had hypertension for almost a decade which time to time raised around 200 mmHg and medications had no benefit on lowering it. The patient also stated that nodules on the body existed from his earliest recollection and he had relatives with neurofibromatosis.

**Figure 1.**
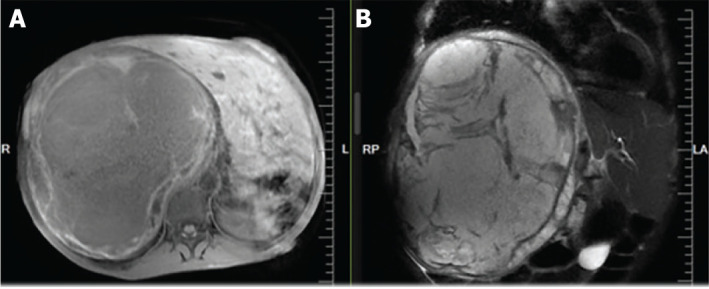
**(A)** Axial post-contrast fat-suppressed T1W image; **(B)** coronal T2W fat-suppressed image that the mass has hypointense weighted heterogeneity in T1 image and hyperintense signaling features in T2 image.

**Figure 2.**
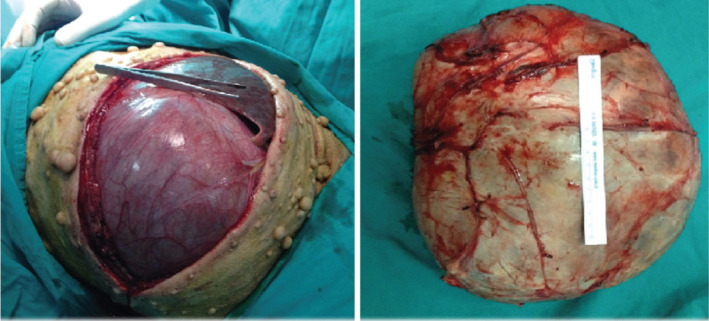
Appearance of the neurofibromas, the café-au-lait spots, and the right lobe of the liver through a subcostal incision in the abdomen, right lobe of the liver pushed to the left side of the abdominal cavity by the mass and composite pheochromocytoma that was surgically removed.

**Figure 3.**
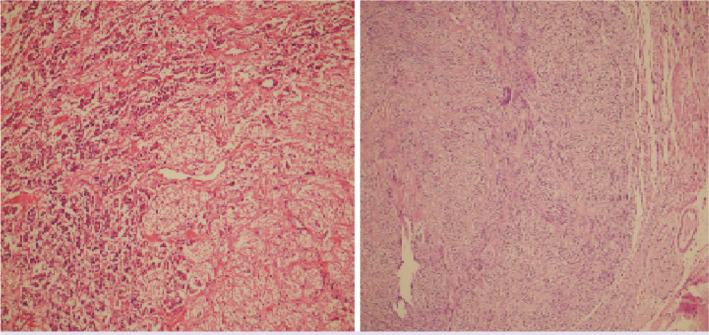
Microscopic view of the tumor with areas of pheochromocytoma on the left side and ganglioneuromas on the right (hematoxylin and eosin stain ×40) and gastrointestinal stromal tumor composed of fibroid cells in bundle structures (hematoxylin and eosin stain ×40).

Mild anemia observed in the biochemistry and hematology examinations (Hb: 8.7 gr/dl and Htc: 28.5%). TA was 170/100 mmHg, and pulse was 106 beats per minute. Additional parameters detected by biochemistry examination are presented as follows; plasma normetanephrine: 7795 pg/ml, plasma metanephrine: 371 pg/ml, sedimentation 127 mm/h, urinary dopamine: 140,490 ug/24 h, urinary adrenalin: 27.3 ug/24 h, urinary noradrenaline: 855 ug/24 s, urinary normetanephrine: 56,658 ug/24 h, urinary metanephrine: 4872 ug/24 h, and vanillylmandelic acid: 284.91 mg/24 h (Tables 1, 2). The gastroscopy of the patient indicated gastritis whereas no feature in colonoscopy was detected. Thyroid USG revealed no other pathology except thyroid parenchymal heterogeneity (thyroiditis?) on radiological examination. There was no feature in the cranial computed tomography (CT) scan. In summary, scoliosis with left side concave, parenchymal bullae in the apical region, parasympathetic centrilobular emphysematous areas, and effusion in the right hemithorax were detected in thorax CT scan examination. A complicated cystic lesion-a vasculature structure pushing liver and kidney aside-between the liver and kidney zone with large-sized contrasting septa was observed in abdominal aortic angiography. In the contrast-enhanced upper abdominal magnetic resonance examination, located between the liver and kidney, a complex cystic mass lesion was noted. The mass had about 28×21 cm in size with thick septations in a distinctly thick irregular wall (Fig. 1). It was also observed that the mass was in hypointense weighted heterogeneity in T1A images and had hyperintense signaling features in T2A images. In the postcontrast series, there was a contrast enhancement in septa and wall (Fig. 1). Based on the endocrine consultation, it was suggested to start Cardura 4 mg (doxazosin mesylate) to the patient who is thought to have pheochromocytoma. Eye consultation revealed multiple Lisch nodules in the iris in the right eye, whereas the zone under the eye was natural. After 10 days of pre-operative (alpha) α-adrenergic blockade preparation, the patient was taken to a surgical operation. Following subcostal incision, an open was made through an adrenal zone and a mass with 30×23 cm in size was removed (Fig. 2). In addition, the area of the nodular structure, with 0.5 cm in diameter, which was found in abdominal exploration in the stomach serosa was also removed. The patient was postoperatively observed in an intensive care unit for 1 day and then transferred the wards. On a post-operative day 2, the oral diet was started and the patient was discharged on the 4^th^ day. On day 6, he was reentered to the hospital with an abdominal pain complaint. Since no pathology was detected and the patient declared no complaint, he was discharged again on the 8^th^ day. Pathologic examinations reported that the removed mass was a composite phaeochromocytoma with ganglioneuromas. The tumor was composed of phaeochromocytoma in the larger spaces and ganglioneuromas in the relatively narrow spaces. Phaeochromocytoma zones were found to be chromogranin (+) and synaptophysin (+) whereas ganglioneuroma zones were found to be S100 (+), neurofilament protein (+), neuron-specific enolase (+), inhibin (-), melanin A (-), CD10 (-), HMB45 (-), and hepar (-). Sustentacular cells were also detected to be S100 (+) (Fig. 3).

**Table 1.** Pre-operative urinary tests of the patient

Hormonal tests-24 h urine	Level	Reference range
Vanillylmandelic acid (mg/h)	284.91/24	0–66
Adrenalin (ug/h)	27.3/24	0–22
Metanephrine	4872/24	0–300
Dopamine	140.490/24	0–500
Noradrenalin	855/24	0–100
Normetanephrine	56.658/24	0–450
5-Hydroxyindole acetic acid	7.08/24	2–9

**Table 2.** Pre-operative blood tests of the patient

Plasma tests	Level	Reference range
Hemoglobin (gr/dl)	8.7	12–18
Hematocrit (%)	28.5	37–52
Alpha-fetoprotein (ng/ml)	2.8	0–8.1
PSA (ng/ml)	0.39	0–3.9
Free PSA (pg/ml)	0.05	0–1
CEA (ng/ml)	2.62	0–4.9
Ca 19.9 (U/ml)	7.94	0–31
Calcitonin (pg/ml)	8.26	0–8.4
Metanephrine (pg/ml)	371	<90
Normetanephrine (pg/ml)	7795	<200
TSH (ul/ml)	1.126	0.55–4.78
sT3 (pg/ml)	1.84	2.3–4.2
sT4 (ng/ml)	0.84	0.7–1.52
Adrenocorticotropic hormone (pg/ml)	34.76	7.2–63.3

PSA: Prostate specific antigen; CEA: Carcinoembryonic antigen; TSH: Thyroid stimulating hormone.

The nodular area removed in gastric serosa was reported as a very low-risk GIST. The tumor was positive in CD117, DOG1, DKA (weakly positive), and negative in S100 and desmin (Fig. 3).

No recurrence was detected in the examinations of post-operative 1^st^, 6^th^ and 12^th^ months periods with normalized biochemical parameters and radiologic examinations. No pathology except gastroesophageal chronic gastritis was detected in gastroscopy.

Written informed consent was obtained from the patient for publication of this case report.

## Discussion

As diagnostic criteria for neurofibromatosis type 1, it was determined that there should two or more of the followings be concomitant; i-6 or more café-au-lait macules (>15 mm post-pubertal), ii-two or more neurofibromas or excess plexiform neurofibroma, iii-axillary and inguinal freckling, iv-two or more Lisch nodules, v-optic glioma, and vi-specific osseous dysplastic lesions. Or else, there should be first-degree relatives with neurofibromatosis type 1 disease that meet these criteria [5]. Neurofibromatosis type 1 disease results from the mutation in the Neurofibromatosis type 1 tumor suppressor gene. The mutation leads to the loss of neurofibromin protein that is responsible for inhibiting the oncogenic Ras activity. As a result of non-inhibited Ras activity, tumor formation is observed in the patient [6].

Pheochromocytoma is a tumor originating from adrenal medulla chromaffin cells and may be seen either sporadic or together with other syndromes. On the other hand, neurofibromatosis type 1 is a disease that can be seen with hereditary pheochromocytomas. The fact that patients with neurofibromatosis lack neurofibromin results in an increased Ras activity which, in turn, activates some of the signaling cascades such as stem cell factor C kit or mitogen-activated kinase pathways.

Neurofibromatosis type 1 patients are at increased risk of developing tumors such as GIST [7]. The incidence of GIST in patients with neurofibromatosis ranges from 3.9% to 25% whereas the possibility of having neurofibromatosis type 1 in GIST is 6% [8]. Different mutations have been detected in GIST formation, in patients with and without neurofibromatosis [9, 10]. That is, activation of the Ras pathway leads to neurofibromatosis type 1 neurofibroma formation, as well as Cajal cell proliferation and ultimately to GIST growth. Moreover, several studies suggest that GISTs in patients with neurofibromatosis type 1 have different molecular pathogenesis than patients with GISTs alone [11].

Our patient had previously been diagnosed with neurofibromatosis. When examined for the mass in the abdomen and hypertension, he was additionally diagnosed with pheochromocytoma. Moreover, the removal of gastric serous nodule kits during surgery and the following diagnostic test revealed that the patient also carries GIST. Therefore, we present this case with a very rare triple diagnosis with neurofibromatosis, pheochromocytoma, and GIST in one patient.

Some authors suggest that pheochromocytoma must be investigated in patients with neurofibromatosis due to the post-operative complications that may arise [12]. This association poses a serious risk of mortality, especially when combined with pregnancy [13].

Although larger masses are also present, pheochromocytomas generally form tissue masses with an average size of 5 cm [14]. However, the size of the mass removed during the surgical operation was 30×28 cm which was larger than the largest one we could identify in 2002 with 27 cm in size. It was also a case of neurofibromatosis type 1 associated with pheochromocytoma [14].

In terms of diagnosis of these three conditions; neurofibromatosis and pheochromocytoma could be clearly diagnosed whereas GISTs are often diagnosed during surgical exploration and pathologic examination of excision material [12, 15, 16]. Although it was possible to make the triple diagnose preoperationally, in our case, it was performed as described in the previous way [17]. Based on reports in the literature, it is safe to state that coming across this triple combination is a quite rare case. Gorgel et al. [4] have mentioned only 15 records to date, including the one introduced in their study. Besides, the pheochromocytoma being in a composite form, made our case even rarer than previously mentioned ones in the literature.

The patient returned to normal life after the operation and there was no serious recurrence of any of the complications in the following year.

According to guidelines, patients with NF1 should have a clinic visit once a year for a clinical evaluation and measurement of blood pressure. The American College of Medical Genetics and Genomics’ recommendation is only patients with NF1 and hypertension, >30 years, pregnant, and/or symptomatic should be evaluated with biochemical and imaging screening [18, 19]. However, recent studies recommend biochemical measurements such as urinary or plasma metanephrines at an early age (10–14 years) and repeating them every 3 years due to consideration of risk of developed catastrophic sequelae on under-recognition of pheochromocytomas/paragangliomas [20]. We decided to have a clinical evaluation and biochemical screening once a year for our patient due to the tendency of frequent surveillance in the literature.

## References

[1] Gutmann DH, Ferner RE, Listernick RH, Korf BR, Wolters PL, Johnson KJ (2017). Neurofibromatosis type 1.. Nat Rev Dis Primers.

[2] Basile U, Cavallaro G, Polistena A, Giustini S, Orlando G, Cotesta D, Petramala L (2010). Gastrointestinal and retroperitoneal manifestations of type 1 neurofibromatosis.. J Gastrointest Surg.

[3] Gutmann DH, Aylsworth A, Carey JC, Korf B, Marks J, Pyeritz RE (1997). The diagnostic evaluation and multidisciplinary management of neurofibromatosis 1 and neurofibromatosis 2.. JAMA.

[4] Gorgel A, Cetinkaya DD, Salgur F, Demirpence M, Yilmaz H, Karaman EH (2014). Coexistence of gastrointestinal stromal tumors (GISTs) and pheochromocytoma in three cases of neurofibromatosis type 1 (NF1) with a review of the literature.. Intern Med.

[5] Neurofibromatosis. (1988). Conference statement.. National Institutes of Health Consensus Development Conference. Arch Neurol.

[6] Fitzgerald PA, Gardner DG, Shoback D (2011). Adrenal medulla and paraganglia.. In: Greenspan’s Basic and Clinical Endocrinology..

[7] Giuly JA, Picand R, Giuly D, Monges B, Nguyen-Cat R (2003). Von Recklinghausen disease and gastrointestinal stromal tumors.. Am J Surg.

[8] Miettinen M, Fetsch JF, Sobin LH, Lasota J (2006). Gastrointestinal stromal tumors in patients with neurofibromatosis 1: a clinicopathologic and molecular genetic study of 45 cases.. Am J Surg Pathol.

[9] Laycock-van Spyk S, Thomas N, Cooper DN, Upadhyaya M (2011). Neurofibromatosis type 1-associated tumours: their somatic mutational spectrum and pathogenesis.. Hum Genomics.

[10] Maertens O, Prenen H, Debiec-Rychter M, Wozniak A, Sciot R, Pauwels P (2006). Molecular pathogenesis of multiple gastrointestinal stromal tumors in NF1 patients.. Hum Mol Genet.

[11] Nemoto H, Tate G, Schirinzi A, Suzuki T, Sasaya S, Yoshizawa Y (2006). Novel NF1 gene mutation in a Japanese patient with neurofibromatosis type 1 and a gastrointestinal stromal tumor.. J Gastroenterol.

[12] Vlenterie M, Flucke U, Hofbauer LC, Timmers HJ, Gastmeier J, Aust DE (2013). Pheochromocytoma and gastrointestinal stromal tumors in patients with neurofibromatosis type I.. Am J Med.

[13] Serleth HJ, Cogbill TH, Gundersen SB (1998). Ruptured pancreaticoduodenal artery aneurysms and pheochromocytoma in a pregnant patient with neurofibromatosis.. Surgery.

[14] Kocakusak A, Sunar H, Asıcı B, Dolap O, Arikan S, Akinci M (2002). Ailesel nörofibramatozis ile beraber görülen dev sessiz feokromasitoma olgusu: Vaka takdimi.. Haseki Tıp Bülteni.

[15] Kramer K, Hasel C, Aschoff AJ, Henne-Bruns D, Wuerl P (2007). Multiple gastrointestinal stromal tumors and bilateral pheochromocytoma in neurofibromatosis.. World J Gastroenterol.

[16] Pan D, Liang P, Xiao H (2016). Neurofibromatosis type 1 associated with pheochromocytoma and gastrointestinal stromal tumors: A case report and literature review.. Oncol Lett.

[17] Ozcinar B, Aksakal N, Agcaoglu O, Tukenmez M, Ozemir IA, Barbaros U (2013). Multiple gastrointestinal stromal tumors and pheochromocytoma in a patient with von Recklinghausen’s disease.. Int J Surg Case Rep.

[18] Ferner RE, Huson SM, Thomas N, Moss C, Willshaw H, Evans DG (2007). Guidelines for the diagnosis and management of individuals with neurofibromatosis 1.. J Med Genet.

[19] Stewart DR, Korf BR, Nathanson KL, Stevenson DA, Yohay K (2018). Care of adults with neurofibromatosis type 1: a clinical practice resource of the American College of Medical Genetics and Genomics (ACMG).. Genet Med.

[20] Al-Sharefi A, Javaid U, Perros P, Ealing J, Truran P, Nag S (2019). Clinical presentation and outcomes of phaeochromocytomas/paragangliomas in neurofibromatosis Type 1.. Eur Endocrinol.

